# Hyperspectral Imaging Combined with Machine Learning Can Be Used for Rapid and Non-Destructive Monitoring of Residual Nitrite in Emulsified Pork Sausages

**DOI:** 10.3390/foods13193173

**Published:** 2024-10-06

**Authors:** Woo-Young Son, Mun-Hye Kang, Jun Hwang, Ji-Han Kim, Yash Dixit, Hyun-Wook Kim

**Affiliations:** 1 Division of Animal Bioscience & Integrated Biotechnology, Gyeongsang National University, Jinju 52828, Republic of Korea; sonwy001223@naver.com (W.-Y.S.); hwangjun1116@naver.com (J.H.); 2 School of Aerospace Engineering, Gyeongsang National University, Jinju 52725, Republic of Korea; kangmh@gnu.ac.kr; 3 Smart Foods, AgResearch, Palmerston North 4410, New Zealand; jihan.kim@agresearch.co.nz (J.-H.K.); yash.dixit@agresearch.co.nz (Y.D.); 4 Department of GreenBio Science, Gyeongsang National University, Jinju 52725, Republic of Korea

**Keywords:** hyperspectral imaging, sodium nitrite, machine learning, pork sausage, real-time monitoring

## Abstract

The non-destructive and rapid monitoring system for residual nitrite content in processed meat products is critical for ensuring food safety and regulatory compliance. This study was performed to investigate the application of hyperspectral imaging in combination with machine learning algorithms to predict and monitor residual nitrite concentrations in emulsified pork sausages. The emulsified pork sausage was formulated with 1.5% (*w*/*w*) sodium chloride, 0.3% (*w*/*w*) sodium tripolyphosphate, 0.5% (*w*/*w*) ascorbic acid, and sodium nitrite at concentrations of 0, 30, 60, 90, 120, and 150 mg/kg, based on total sample weight. Hyperspectral imaging measurements were conducted by capturing images of the cross-sections and lateral sides of sausage samples in a linescan mode, covering the spectral range of 1000–2500 nm. The analysis revealed that higher nitrite concentrations could influence the protein matrix and hydrogen-bonding capacities, which might cause increased reflectance at approximately 1080 nm and 1280 nm. Machine learning models, including XGBoost, CATboost, and LightGBM, were employed to analyze the hyperspectral data. XGBoost demonstrated the best performance, achieving an *R*^2^ of 0.999 and a root mean squared error of 0.095, highlighting its high predictive accuracy. This integration of hyperspectral imaging with advanced machine learning algorithms offers a non-destructive and real-time method for monitoring residual nitrite content in processed meat products, noticeably improving quality control processes in the meat industry. Additionally, real-time implementation in industrial settings could further streamline quality control and enhance operational efficiency. Further research should focus on validating these findings with larger sample sizes and more diverse datasets to ensure robustness.

## 1. Introduction

Nitrites play a pivotal role in the formation of desirable quality attributes in cured meat products, including color development, flavor enhancement, oxidative stability, and microbial safety, which are essential for consumer acceptance and satisfaction [1]. In a 2021 survey conducted by Citizens United for Consumer Sovereignty (CUCS) in Korea, it was reported that approximately 92% of processed meat products contain nitrite salts, reflecting its extensive use in the meat-processing industry. In cured meat products, it has been well documented that 30–60% of the added nitrite reacts with muscle proteins and lipids, while 5–20% remains as residual nitrite in the final product [2]. The persistence of residual nitrite raises significant health concerns due to its ability to reach with secondary amines or biogenic amines, forming carcinogenic nitrosamines, which are particularly linked to liver toxicity and hepatocarcinogenesis [3]. The International Agency for Research on Cancer (IARC) has classified processed meats containing nitrites as Group 1 carcinogens [4], reinforcing the public health implications related to nitrite consumption. With increasing consumer awareness of these risks, there is also a growing demand within the meat-processing industry for advanced monitoring systems capable of accurately quantifying and controlling residual nitrite levels in processed meat products.

Given the well-documented health risks associated with nitrite consumption, many countries have implemented stringent regulations to limit residual nitrite levels in processed meat products. In the case of Korea, the Ministry of Food and Drug Safety enforces a maximum allowable residual nitrite concentration of 70 mg/kg in meat products [5]. To ensure regulatory compliance, random sampling and monitoring of commercial products is commonly conducted. The Korean Food Code outlines standardized methodologies for measuring residual nitrite, with colorimetric and ion chromatography techniques being the most widely employed [6]. The colorimetric method involves the extraction of nitrite ions, which then undergo a diazotization reaction in acidic conditions. The resulting diazonium compound is coupled with *N*-(1-naphthyl) ethylenediamine to form an azo dye, which is quantified spectrophotometrically [7]. In comparison, ion chromatography entails the extraction of nitrite ions in an aqueous solution, followed by purification and separation through an anion exchange column, with detection using a suppressor–conductivity detector [8]. While these analytical methods are well established and reliable, they present certain limitations, such as sample destruction, labor-intensive procedures, and the potential for variability in results due to operator skill.

Hyperspectral imaging (HSI) technology enables the acquisition of vast amount of data by capturing hundreds of consecutive spectral bands across a broad range of wavelengths. This rich spectral information provides a solid foundation for the development of accurate classification and clustering algorithms for similar sample types [9]. The primary advantages of HSI systems include real-time data acquisition, operational simplicity, high accuracy, reproducibility, and long-term durability, making them highly applicable to industrial environments [10]. Recent research has increasingly focused on utilizing HSI for the non-contact, non-destructive analysis of food products, allowing the identification of substance-specific spectral characteristics at the molecular level [11,12,13]. This capability offers a promising approach for the precise characterization of food components. As interest grows in developing prediction models for food compounds and properties, machine learning, a key area within artificial intelligence, has emerged as a powerful tool for processing large and complex datasets. Machine learning algorithms enable systems to learn autonomously from data, adapt to new information, and make data-driven decisions with minimal human intervention. With advancements in computational power and programming techniques, machine learning has seen rapid progress, particularly in food science and industry, where it enhances both process efficiency and predictive accuracy [14,15]. Recent studies have underscored the superior performance of machine learning algorithms over traditional methods, such as partial least squares regression (PLSR), especially when handling hyperspectral data, since machine learning algorithms offer greater flexibility, accuracy, and robustness, particularly when dealing with nonlinear relationships, performing feature selection, and managing large-scale or noisy datasets [16].

Given the increasing industrial demand for non-destructive and rapid monitoring of residual nitrite levels in processed meat products, traditional methods are often inadequate for providing real-time assessment. This implies the need for advanced, continuous systems capable of directly measuring residual nitrite levels on conventional conveyor belt systems in the meat-processing industry. The implementation of such systems would not only enhance product safety but also streamline quality control processes, ensuring consistent compliance with regulatory standards while addressing rising consumer expectations. Although previous studies have explored the development of prediction models for residual nitrite content in processed meats using hyperspectral imaging [17], the potential of integrating machine learning into these models remains underexplored. In this regard, it could be hypothesized that hyperspectral imaging can detect unique spectral characteristics associated with residual nitrate in processed meats, such as emulsified pork sausages, which could then be utilized for the accurate quantification of residual nitrite using machine learning algorithms. Therefore, this study aims to evaluate the feasibility of using a hyperspectral imaging model, enhanced by machine learning algorithms, for the quantitative determination of residual nitrite content in emulsified pork sausages.

## 2. Materials and Methods

### 2.1. Sample Preparation

Fresh pork ham and back fat (A fat) were purchased from a local food market (M-12S, Hankook Fujee Industries, Hwaseong, Republic of Korea), vacuum-packaged in PE/NY bags for transport, and stored under refrigeration until use. Surface fat and connective tissue were manually removed from the pork ham and back fat. The trimmed pork ham was then ground using a meat grinder (M-12S, Hankook Fujee Industries, Gyeonggi-do, Republic of Korea) equipped with a 6 mm plate. The emulsified pork sausages were formulated with 60% (*w*/*w*) ground pork, 20% (*w*/*w*) back fat, and 20% (*w*/*w*) ice. The formulation also included 1.5% (*w*/*w*) sodium chloride, 0.3% (*w*/*w*) sodium tripolyphosphate, 0.5% (*w*/*w*) ascorbic acid, and sodium nitrite at serial concentrations of 0, 30, 60, 90, 120, and 150 mg/kg, based on the total sample weight. The main ingredients and food additives were emulsified in a bowl cutter (C6, SIRMAN SpA, Curtarolo, Italy), and the meat emulsion was stuffed into collagen casings (Ø25, Fcase, Jawornik, Poland) using a sausage stuffer (Korea Times Square Co., Chungcheongnam-do, Republic of Korea), with an average sausage weight of 35.5 ± 3.7 g. The raw sausages were then heated in a water bath (JSIB-22T, JS Research Inc., Hwaseong, Republic of Korea) set at 80 °C until the core temperature reached 71 °C, followed by cooling at 10 °C for 20 min.

### 2.2. Physicochemical Analysis of Emulsified Pork Sausage

#### 2.2.1. Cooking Loss

The cooking loss of the emulsified pork sausages was calculated using the following equation: cooking loss (%) = ((weight before cooking (g) − weight after cooking (g))/weight before cooking (g)) × 100.

#### 2.2.2. Color Analysis

The surface color of the cross-sections of the emulsified pork sausages was measured using a Chroma Meter CR-400 (Konica Minolta, Inc., Tokyo, Japan) equipped with a 2° observer and D_65_ illuminant source. The colorimeter was calibrated using the manufacturer’s calibration plate (CIE L* value, 93.01; CIE a* value, −0.25; CIE b* value, +3.50). The results for CIE a* (redness) were recorded.

#### 2.2.3. Residual Nitrite Content

The residual nitrite ion content in the emulsified pork sausages was determined using a modified diazotization method described by Sreekumar et al. [7]. This method involves the diazotization of sulfanilamide by nitrite ions under acidic conditions, followed by coupling with *N*-(1-naphthyl) ethylenediamine to form an azo dye. An ammonium acetate buffer was prepared by dissolving 100 g of ammonium acetate in 900 mL of distilled water and adjusting the pH to 9.0 using a 10% (*w*/*v*) ammonia solution, and the solution was diluted with distilled water to obtain a final volume of 1000 mL. For the sulfanilamide solution, 0.5 g of sulfanilamide was dissolved in 100 mL of hydrochloric acid by heating the mixture. To prepare the *N*-(1-naphthyl) ethylenediamine solution, 0.12 g of *N*-(1-naphthyl) ethylenediamine hydrochloride was dissolved in 100 mL of distilled water and stored in a brown bottle. The sausage sample (10 g) was homogenized in distilled water at 80 °C and mixed with 10 mL of 0.5 N sodium hydroxide solution and 10 mL of 12% zinc sulfate solution. The mixture was heated in a water bath at 80 °C for 20 min and rapidly cooled in cold water (10 °C). The mixture was diluted to a final volume of 200 mL with the ammonium acetate buffer and distilled water, centrifuged at 4000 rpm for 10 min, and filtered through the Whatman No. 4 filter paper to obtain a clear filtrate as the sample solution. For the assay procedure, 20 mL of the sample solution was mixed with 1 mL of the sulfanilamide solution and 1 mL of the *N*-(1-naphthyl) ethylenediamine solution. Each mixture was diluted with 25 mL of distilled water, reacted for 10 min, and measured for absorbance at 540 nm. The nitrite ion content in the sample solution and blank solution was determined using a standard curve, and the residual nitrite content was calculated as follows: nitrite ion (g/kg) = A/S × 1/100, where A is the difference in nitrite content between the sample solution and blank solution and S is the sample weight (g).

### 2.3. Hyperspectral Imaging Data Acquisition

**Figure 1:**
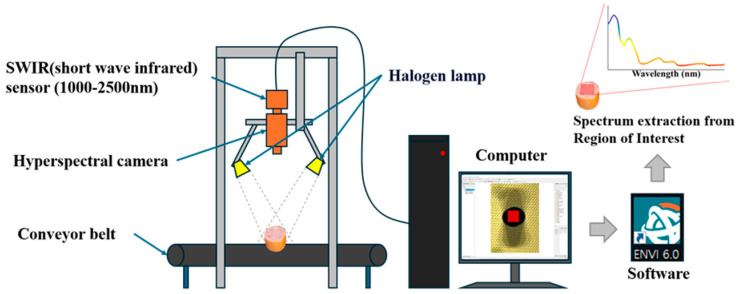
The schematic diagrams of the main components of the hyperspectral imaging systems.

The schematic diagram of the main components of the hyperspectral imaging system used in this study is presented in Figure 1. The hyperspectral imaging (HSI) camera (SWIR, SPECIM, Oulu, Finland) was used in linescan mode. The spectral range of the camera used was 1000–2500 nm. The HSI system covered 288 continuous spectral bands, with a spectral interval of 5.62 nm. The exposure time was set within a range of 0.1–20 ms. The focal length and frame rate were set at 9.2 mm and 450 fps, respectively. The illumination was provided by six halogen lamps (MR16, 12V, 20W, 210Lm, Osram, Munich, Germany) positioned at a 45° angle to the focusing area. Measurements were conducted in a darkroom with a relative humidity of 30% and an ambient temperature of 20 °C. For measurements, samples were prepared by cutting the middle part of the pork sausages into 3 cm-thick cross-sections. The cross-sections and the lateral sides of the sausages were imaged; 20 samples per treatment were placed on a conveyor belt at 4 cm intervals, both vertically and horizontally, to capture images of the cross-sections and lateral sides. The spatial resolution and pixel size of the images were 384 pixels and 24 × 24 μm, respectively. All hyperspectral images were processed and analyzed using ENVI software (The Environment for Visualizing Images, version 6.0, SELAB, Seoul, Republic of Korea). A 25 × 25 (625) pixel region of interest (ROI) containing the center pixel of the sample was selected, and the mean spectrum of the ROI was set as the spectrum of each sample. For data processing, the raw spectral data were automatically normalized by dividing each sample spectrum by a reference spectrum obtained from a white standard, minimizing the influence of external factors during image acquisition. For background removal, the central 625 pixels of each hyperspectral image, corresponding to the core region of the sausage, were selected to effectively eliminate any background interference. The spectral data for each sample were averaged from these central pixels, ensuring that only relevant information from the sample was included, with no background data used in the analysis.

### 2.4. Machine Learning Model Development

To predict residual nitrite ion content in the cross-sections and sides of emulsified pork sausages, an advanced ensemble technique using a gradient boosting framework was employed. This framework utilized the hyperspectral spectrum range of 1000–1500 nm, which exhibited significant differences in the measured residual nitrite ions. Specifically, the algorithms CATboost, LightGBM, and XGBoost were employed [18,19,20], as these sequentially combine weak learners to form a strong predictive model. XGBoost constructs tree structures using Classification And Regression Tree (CART) and employs a level-wise approach for splitting leaves, unlike LightGBM with a leaf-wise approach. This technique is widely used in the data science community and maximizes prediction performance and training speed through parallel processing and regularization techniques. While it offers high performance and stability, hyperparameter tuning can be complex. Each algorithm was implemented using its respective library, and functionalities for data splitting and model performance evaluation were implemented using the scikit-learn library. Among the hyperparameters used in the library (Table 1), the learning rate was set to 0.01 to balance the model’s learning speed and performance, thereby optimizing the model’s effectiveness. The tree depth was set to 3 for the experiments. In addition, the data used in all machine learning models were divided into training and predictive models at a ratio of 4:1 on a total scale (100 sets per treatment).

### 2.5. Model Performance Evaluation

The accuracy of the machine learning model predicting the residual nitrite ion content in emulsified pork sausages was evaluated using the root mean squared error (RMSE) and the coefficient of determination (*R*^2^). RMSE is defined as the square root of the mean squared error (MSE), which is a commonly used loss function. MSE was calculated by dividing the sum of the squared residuals of each data point by the number of data points. Since MSE can be overly sensitive due to the squaring of residuals, RMSE was used instead. The coefficient of determination (*R*^2^) metric indicates how well the predicted values (t) explain the variability of the input values. A value closer to 1 signifies a stronger positive correlation. Both metrics were computed in Python using the scikit-learn library, specifically with the mean_squared_error function for RMSE and the model.score function for *R*^2^.

### 2.6. Statistical Analysis

All data were expressed as mean ± standard error (S.E.). Statistical analyses were performed using SPSS Statistics (Version 18.0, IBM, Armonk, NY, USA). Significance was tested using one-way analysis of variance (one-way ANOVA) and Duncan’s multiple range test with a significance level of *p* < 0.05. All hyperspectral images were processed and analyzed using ENVI software (The Environment for Visualizing Images, version 6.0, SELAB, Seoul, Republic of Korea). Machine learning algorithms were implemented in Python using the scikit-learn library.

## 3. Results and Discussion

### 3.1. Cooking Loss, Redness, and Residual Nitrite Content of Emulsified Pork Sausage

The cooking loss, redness, and residual nitrite content of emulsified pork sausages formulated with varying levels of sodium nitrite are shown in Table 2. Cooking loss, which reflects the loss of moisture and fat during the heating process, can significantly alter the relative proportions of major components in processed meat products [21]. These components, particularly moisture and fat, are known to influence surface reflectance properties in hyperspectral imaging of meat products [22]. In this study, the samples showed no significant differences in cooking loss (*p* > 0.05), irrespective of the sodium nitrite concentrations added. This consistency in cooking loss indicates that the proportions of major components (e.g., moisture and fat), which can affect hyperspectral imaging data, remained largely unchanged across the different sodium nitrite treatments. Consequently, the results suggest that cooking loss is unlikely to have a substantial impact on the hyperspectral imaging data obtained from these samples. Thus, variations in sodium nitrite levels appear not to interfere with the spectral properties of the sausages, ensuring the consistency of hyperspectral data analysis.

As expected, CIE a* (redness) of the cross-sections of emulsified pork sausages increased as the level of sodium nitrite increased. Nitrite is a common colorant used to guarantee the desirable pink color in cured meat products [23], and it has been well documented that the addition of nitrites results in a dose-dependent increase in the redness of processed meat products [24]. In this study, however, the addition of sodium nitrite above 120 ppm resulted in similar redness of emulsified pork sausages (*p* > 0.05). Some previous studies have reported similar results, showing no difference in the redness of processed meat products above certain levels of sodium nitrite [24]. These results may indicate that simple spectroscopic information in the visible light region could be insufficient to predict the residual nitrite content in processed meat products.

The residual nitrite content of emulsified pork sausage formulated with 0 to 150 ppm of sodium nitrite ranged from 0.14 to 21.35 ppm, respectively, accounting for 13.5% to 17.1% of the added amounts. According to Pegg [2], 30–60% of the nitrite added to meat products reacts chemically with muscle proteins and lipids, while the remaining 5–20% persists as nitrite and 1–10% as nitrate in the final product. Our results were consistent with this previous suggestion [2]. Moreover, there was a significant difference in residual nitrite content between the set nitrite ranges (in 30 ppm intervals). Thus, it could be expected that these samples are suitable for obtaining reliable hyperspectral data based on the significant differences in residual nitrite content.

### 3.2. Spectral Characteristics of Emulsified Pork Sausage

**Figure 2:**
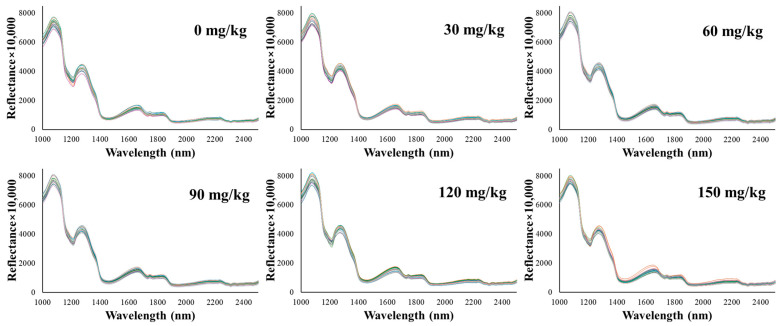
Spectra (1000–2500 nm) of the cutting surface of emulsified pork sausages with different levels of added sodium nitrite.

**Figure 3:**
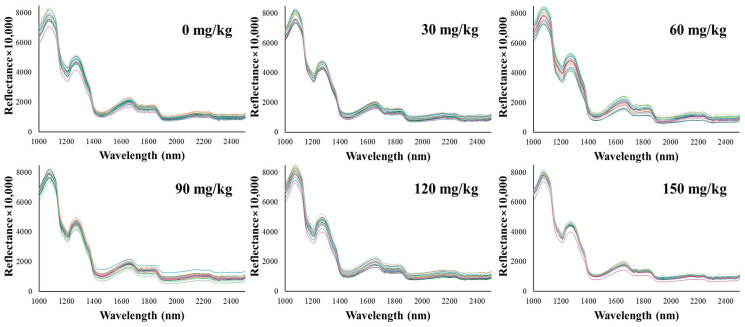
Spectra (1000–2500 nm) of the lateral surface (on casing) of emulsified pork sausages with different levels of added sodium nitrite.

The analysis of hyperspectral data from both cross-sectional (Figure 2) and lateral (Figure 3) views of emulsified pork sausages revealed distinctive spectral characteristics associated with varying sodium nitrite levels. Specifically, the spectral peaks within the 1000–2500 nm range displayed notable absorption features, which could be linked to the chemical composition and structural properties of the sausage samples. In this study, two major absorption peaks were observed at approximately 1080 nm and 1280 nm. The reflectance at approximately 1080 nm and 1280 nm increased with increasing sodium nitrite levels, suggesting a strong correlation between nitrite concentration and changes in the protein structure within the meat matrix [24].

Sodium nitrite is known to play a key role in stabilizing proteins, particularly through interactions with sulfhydryl groups and the formation of nitrosylated compounds [25]. This stabilization affects the protein’s secondary structure, potentially enhancing the strength of hydrogen bonding in the protein matrix [26]. Such structural changes could be responsible for the observed spectral behavior, especially at the noted wavelengths, as these changes reflect alterations in N–H stretch overtones. Previously, hyperspectral imaging could capture the diffusion of sodium nitrite within the sausage matrix, suggesting its potential to detect changes in water-binding capacity and protein stabilization [27]. Our observation was consistent with findings from Gong et al. [28], who also reported similar spectral behavior in meat products with varying nitrite concentrations. The observed increase in reflectance at these specific wavelengths may be attributed to enhanced water-binding capacity facilitated by nitrite, affecting the O–H and N–H stretch overtones, which reflect changes in water content and protein conformation [29,30]. In addition, Kucha et al. [31] demonstrated that nitrite ions can interact with myofibrillar proteins and promote cross-linking, thereby enhancing the water-binding capacity in processed meats, which can be correlated with these structural changes at the molecular level through changes in reflectance. Previously, Tantinantrakun et al. [17] identified absorption peaks at 1208 nm and 1460 nm, which they attributed to food additives affecting C–O, C–H, and N–H bonds. These disparities in peak positions and their corresponding attributions underscore the specific spectral responses of different meat types (pork versus chicken) and highlight the distinctive role of nitrite in pork sausages compared to other additives in chicken products. This finding emphasizes the importance of understanding the unique interactions between meat composition and specific additives, as these interactions can vary significantly between different types of processed meats.

**Figure 4:**
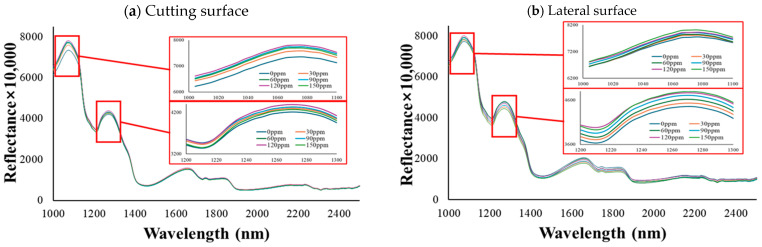
Average spectra (1000–2500 nm) of the cutting surface (**a**) and lateral side (**b**) of emulsified pork sausages with different levels of sodium nitrite. The spectrum was magnified to focus on the regions of interest around 1080 nm (1000–1100 nm) and 1280 nm (1200–1300 nm). These regions were selected due to their evident changes in the peaks, providing key insights into the specific interactions within the sample matrix.

Furthermore, the spectral data obtained from both cross-sectional and lateral views of the sausages exhibited consistent trends (Figure 4). Both orientations showed similar increases in reflectance at the identified peaks, indicating a homogenous distribution of nitrite-induced chemical changes within the sausages. This uniformity across different views is critical for the application of hyperspectral imaging as a reliable tool for meat quality assessment, ensuring that accurate and consistent measurements can be obtained regardless of product orientation. Overall, the findings from this study contribute to the expanding body of knowledge on the role of nitrite in processed meat products, demonstrating the efficacy of hyperspectral imaging as a non-destructive method for monitoring chemical changes. The ability to detect such changes reliably across different viewing angles further highlights the potential for hyperspectral imaging to be implemented in industrial settings, where rapid and precise quality control is essential.

### 3.3. Nitrite Prediction by Machine Learning Model Using a Wavelength of 1000–1500 nm

The development and application of machine learning models for predicting nitrite levels in emulsified pork sausages focused on the wavelength range of 1000–1500 nm. This wavelength range was specifically selected due to the spectral features changed by sodium nitrite concentration, as supported by earlier research, such as Zhu et al. [32], which employed a broader 900–1700 nm range for nitrite prediction in ham sausages. Previous studies have selected effective wavelengths for predictive modeling using hyperspectral spectra from sausage samples, but these approaches may have limited applicability across commercial sausage products [33]. Moreover, machine learning models can integrate the entire dataset without pre-selecting specific wavelengths, potentially improving their generalizability. In this study, advanced machine learning algorithms, including XGBoost, CATboost, and LightGBM, were employed to predict residual nitrite content with high accuracy. The strategic selection of the 1000–1500 nm range optimized the use of spectral data while maintaining computational efficiency, which is crucial for real-time predictive modeling.

The results demonstrated strong predictive capabilities across all models, with training accuracies exceeding 0.99 and test accuracies ranging from 0.988 to 0.999 (Table 3). Among the algorithms evaluated, XGBoost exhibited the highest performance, achieving an R^2^ value of 0.999 and an RMSE of 0.095. This exceptional predictive accuracy underscores the model’s effectiveness in capturing the relationship between spectral data and residual nitrite concentration, establishing XGBoost as a highly reliable tool for nitrite prediction in processed meat products. Furthermore, the use of specific wavelength range contributed to the model’s computational efficiency, reducing the processing load while preserving prediction accuracy. This feature is particularly valuable for industrial applications, where rapid and accurate quality control is critical. In comparison, earlier work by Zhu et al. [32] utilizing partial least squares (PLS) regression models over a broader wavelength range reported lower R^2^ values and higher RMSEs, indicating that machine learning approaches, particularly XGBoost, offer superior performance for this type of predictive task. While PLS models are effective for nitrite prediction, the results from Zhu et al. [32] suggest that machine learning algorithms are better suited for capturing the complexity of hyperspectral data and the nonlinear relationships between spectral features and nitrite content.

Moreover, this study highlights the potential of combining hyperspectral imaging with advanced machine learning techniques to achieve real-time, highly accurate predictions. The application of XGBoost, an ensemble learning technique, emphasizes the advantages of model aggregation, which enhances overall predictive accuracy compared to traditional regression methods. This methodology marks a significant advancement in the field of food quality assessment, showcasing the value of machine learning over classical regression techniques used in prior research [32]. In addition, the focused 1000–1500 nm wavelength range used in this study offers potential advantages over broader spectral ranges. This selected range is more adaptable to different hyperspectral imaging systems and reduces the variability introduced by differences in sensor technology, ensuring greater model robustness across various industrial applications. This adaptability is crucial in the food industry, where rapid and reliable quality control is essential for maintaining product standards. Furthermore, it is expected that each pixel of the hyperspectral image can be predicted using a highly predictive machine learning model and remapping it to see the residual nitrite distribution of sausage samples.

Recently, the hyperspectral imaging technique for food component analysis has been proposed as a powerful analytical method, offering several advantages over conventional approaches [34]. Traditional methods for analyzing residual nitrite ions in processed meat products are destructive, labor-intensive, and time-consuming. In contrast, the hyperspectral imaging approach determined in this study could provide the potential for non-destructive and rapid prediction models that could be effectively implemented in industrial applications. Additionally, the integration of advanced machine learning algorithms may enhance the accuracy and sensitivity of these models [34]. The development of such systems presents several benefits, including real-time product monitoring, a reduction in experimental errors, the creation of robust databases, and the elimination of chemical reagent use, making the process more efficient and environmentally sustainable [35].

## 4. Conclusions

This study successfully demonstrated the application of hyperspectral imaging combined with machine learning algorithms for the non-destructive prediction of residual nitrite levels in pork sausages. The spectral analysis, focused on the near-infrared region (1000–2500 nm), identified key absorption peaks at 1080 nm and 1280 nm, which were associated with nitrite-induced alterations in water-binding capacity and protein stability. Among the machine learning models evaluated, XGBoost exhibited the highest performance, achieving an R^2^ value of 0.999 and an RMSE of 0.095, indicating its exceptional accuracy in predicting residual nitrite content. These findings imply the potential of integrating hyperspectral imaging with machine learning to enhance real-time quality control in the meat industry, providing a rapid and non-destructive alternative to conventional analytical methods. Further research should focus on validating these results with larger datasets to ensure robustness and generalizability. Moreover, additional exploration of the underlying molecular interactions that drive the spectral changes could lead to more refined prediction models, expanding the utility of hyperspectral imaging and machine learning for widespread use across the food industry. For industrial applications, it will also be necessary to establish a stable hyperspectral measurement system capable of obtaining consistent data within the same product group and applicable to a variety of product categories.

## Figures and Tables

**Table 1:** The hyperparameter settings of the specific model.

Models	Hyperparameters	Value	Models	Hyperparameters	Value	Models	Hyperparameters	Value
CATboost	interations	1200	LightGBM	n_estimators	1200	XGBoost	interations	1200
	learning_rate	0.01		learning_rate	0.01		learning_rate	0.01
	depth	3		max_depth	3		depth	3

**Table 2:** The color, cooking loss, and residual nitrite of emulsified pork sausages formulated with different levels of sodium nitrite.

Traits	0 mg/kg		30 mg/kg		60 mg/kg		90 mg/kg		120 mg/kg		150 mg/kg	
	Mean	SD ^(1)^	Mean	SD	Mean	SD	Mean	SD	Mean	SD	Mean	SD
Cooking loss (%)	98.78	0.06	98.67	0.23	98.77	0.07	98.74	0.15	98.54	0.34	98.72	0.19
CIE a* (redness)	2.41 ^e^	0.17	6.19 ^d^	0.42	7.68 ^c^	0.31	8.78 ^b^	0.13	9.17 ^ab^	0.02	9.43 ^a^	0.12
Residual nitrite content (mg/kg)	0.14 ^f^	0.1	5.08 ^e^	0.64	8.67 ^d^	0.71	12.27 ^c^	0.99	18.8 ^b^	1.15	21.35 ^a^	1.25

^(1)^ SD: standard deviation. ^a–f^ Means sharing the same letter within a row are not significantly different at *p* < 0.05 by Duncan’s multiple range test.

**Table 3:** Machine learning model performances for predicting sodium nitrite content based on 1000–1500 nm wavelengths.

Measurement Site	Machine Learning Algorithms	Total Set	Calibration Set			Prediction Set		
			Number of Sample	R^2^C	RMSEC	Number of Sample	R^2^P	RMSEP
Cross-section	CATboost	118	95	0.998	0.247	23	0.997	0.404
	LightGBM	118	95	0.992	0.641	23	0.992	0.851
	XGBoost	118	95	0.999	0.001	23	0.999	0.236
Side	CATboost	118	95	0.998	0.034	23	0.988	0.086
	LightGBM	118	95	0.99	0.084	23	0.99	0.125
	XGBoost	118	95	0.999	0.001	23	0.999	0.095

R^2^C = coefficient of determination in calibration; RMSE = root mean square error in calibration; R^2^P = coefficient of determination in prediction; RMSEP = root mean square error in prediction; CATboost = categorical boost; LightGBM = light gradient boosted machine; XGBoost = extreme gradient boosting.

## Data Availability

The original contributions presented in the study are included in the article, further inquiries can be directed to the corresponding author.
